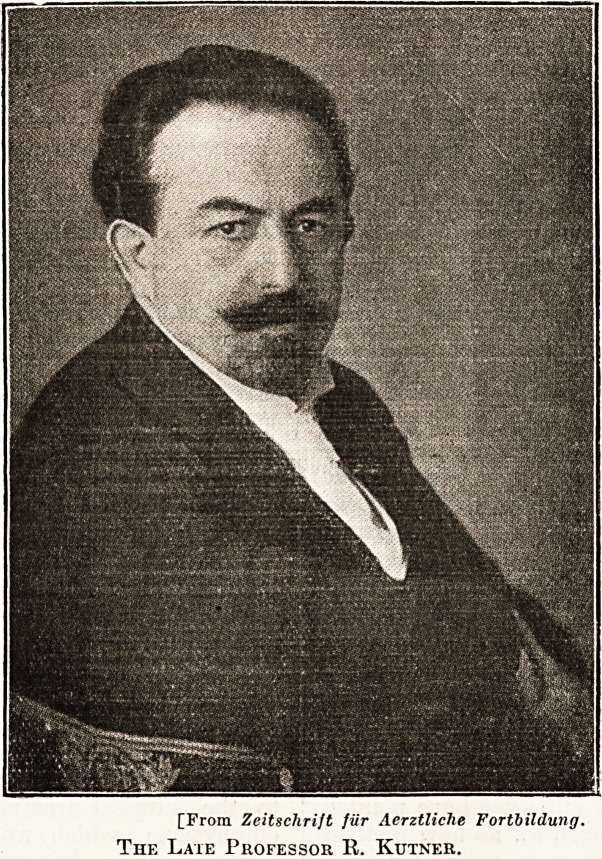# A Pioneer of Post-Graduate Teaching: A Personal Appreciation of Professor R. Kutner

**Published:** 1913-10-25

**Authors:** 


### A PIONEER OF POST-GRADUATE TEACHING.

### A Personal Appreciation of Professor R. Kutner.

Unexpectedly, on the evening of October 5, died at Berlin Professor Robert Kutner, Director of the Kaiserin Friedrich Haus. To readers of The Hospital his name is familiar enough, for we have regularly given details of the progress made by the committee which he established and which has done so much for post-graduate education in Europe.

The news of his death will come as a great blow to all connected with the movement, and will be felt as a personal sorrow by the many medical men who have been privileged to enjoy his friendship, and who found in him a staunch and loyal colleague, a broad-minded, liberal-opinioned man, and a cheery, genial, ever optimistic friend.

The writer became acquainted with Dr. Kutner while engaged in investigating, as Special Commissioner for The Hospital, the progress of hospital work in Germany. A chance visit to the Luisenplatz Institution brought him into contact with the director. At that time the Kaiserin Friedrich Haus was in its pioneering stage; Kutner, with an energy and thoroughness which German workers alone seem to possess, had gathered the diverse strands of the post-graduate movement from all parts of the civilised world in an attempt to braid them together into one thick rope, strong enough to resist local strains and yet pliant enough to adapt itself to necessary inequalities. In an interview lasting the best part of a morning, he explained his ideals, giving and extracting information in that delightful conversational manner which made him so charming a talker and so appreciative a listener. Immensely interested in what had been done in England, he expressed his regret that he had never been able to find time to visit the Chenies Street Polyclinic, with whose consultative system he was in full accord, nor the post-graduate courses at Edinburgh and Oxford. When the writer left Berlin he continued the interchange of ideas then begun by letters which from time to time gave full particulars of the movement. Kutner's work on behalf of the movement has been well recognised. In his own country he was honoured by the Kaiser; he was awarded a Commandership of the Victorian Order; and America, having no such distinctions to offer him, gave him, what he perhaps honoured more, the most ample recognition of his work by graduate centres in the States. The energy that he expended, the zeal and enthusiasm which he showed, were given at the sacrifice of much personal comfort and professional prospects. For had he stuck to his speciality, there is little doubt that Kutner would have made a name for himself in another department. Born in 1867, the son of a provincial practitioner, Kutner studied medicine at Berlin, Kiel, and Freiburg, and then occupied himself with special work in urology under Nitze, Dittel of Vienna, and Guyon of Paris. In 1891 he completed the State examination, and the following year he started practice as a urologist at Berlin. Already as a young student he had thought about the practicability of initiating post-graduate courses in various specialities. The need for such instruction was generally felt, and when the sympathy of Althoff and von Bergmann had been enlisted, the start was made with the fine movement which was to culminate, some years later, in the establishment of the Kaiserin Friedrich Haus. Kutner threw himself heart and soul into the movement; indefatigable and enthusiastic, he worked literally day and night to get progress. The plans were drawn up under his direct supervision; the syllabus was arranged by him, the lecturers were personally secured by his efforts. Personal service he gave to the full, ungrudging, and often single-handed. In his own words "the knowledge that the movement was appreciated, and that the work benefited the profession," was recompense enough. He established the *Zeitschrift für Aerztliche Fortbildung*, which is to-day one of the most useful and authoritative of the many German medical papers. His correspondence must have been immense, for he was at all times prepared to give advice to the many applicants, German and foreign, who either by letter or in person came to him for help in prosecuting their studies on the Continent. Amidst all he continued his interest in his own speciality, and won some reputation in it, though his work at the Kaiserin Friedrich Haus prevented him from doing much hospital practice. His finest monument is the splendid institution of which he was the director, the paper that he founded and for many years so ably edited, and the appreciation which hundreds of his colleagues, who through his indirect efforts were enabled to improve their professional knowledge at Continental centres, must and do feel for the self-sacrificing enthusiasm which prompted him to be a pioneer in a department so long left fallow.

[From *Zeitschrift für Aerztliche Fortbildung*.

The Late Professor R. Kutner.

## Figures and Tables

**Figure f1:**